# Retraction: Prevalence of stress and its determinants among residents enrolled in China Standardized Training Program for Resident Doctor (C-STRD) program: A cross-sectional study

**DOI:** 10.1371/journal.pone.0219653

**Published:** 2019-07-09

**Authors:** The PLOS ONE Editors

Following publication of this [1] article, concerns were noted about the enrolment of participants and the level of ethical oversight for this research. The article states “The current study has been approved by the ethics committee of Shanghai Chang Zheng Hospital,” however the authors were unable to provide ethics approval documentation or any confirmation from their institution to support this statement. The authors have explained that they did not seek written informed consent from study participants due to associated costs but that participation was voluntary.

Furthermore, the affiliation listed for the corresponding author on the published article is incorrect. The corresponding author is affiliated with Department of Neurosurgery, Shanghai Chang Zheng Hospital affiliated to China Second Military Medical University. The author explained that they were affiliated to Rutgers University from January-August 2016, during preparation of this manuscript.

Owing to unresolved concerns about whether this work received the necessary ethical approval prior to its initiation, the *PLOS ONE* Editors retract this article.

The authors did not respond to the retraction decision.
